# Drug Combination in Cancer Treatment—From Cocktails to Conjugated Combinations

**DOI:** 10.3390/cancers13040669

**Published:** 2021-02-07

**Authors:** Yosi Gilad, Gary Gellerman, David M. Lonard, Bert W. O’Malley

**Affiliations:** 1 Department of Molecular and Cellular Biology, Baylor College of Medicine, Houston, TX 77030, USA; yosi@bcm.edu; 2 Department of Chemical Sciences, Ariel University, Ariel 40700, Israel; garyg@ariel.ac.il

**Keywords:** chemotherapy, drug combination, targeted drugs, the history of chemotherapy, drug conjugates

## Abstract

**Simple Summary:**

Chemotherapy is a key modality in today’s practice of clinical oncology. The potential for systemic treatment of cancer patients with cytotoxic agents was discovered in the mid 20th century and its major shortcomings namely, off target toxicity and resistance to treatment, were apparent from the very beginning. These limitations pushed the scientific and medical communities to search for improvements, which as a final result, shaping modern research and clinical practice in the field. For minimizing off target toxic effects, drug discovery efforts became more focused on targeted therapies and on combinations of anti-cancer drugs in order to overcome the resistance problem. Here we outline the evolution of chemotherapy from its beginning as a single-agent based therapy to poly-drug treatment that involves targeted drugs, and discuss the concept of drug-conjugation based treatment as a strategy for further optimization of treatment regimes.

**Abstract:**

It is well recognized today that anticancer drugs often are most effective when used in combination. However, the establishment of chemotherapy as key modality in clinical oncology began with sporadic discoveries of chemicals that showed antiproliferative properties and which as a first attempt were used as single agents. In this review we describe the development of chemotherapy from its origins as a single drug treatment with cytotoxic agents to polydrug therapy that includes targeted drugs. We discuss the limitations of the first chemotherapeutic drugs as a motivation for the establishment of combined drug treatment as standard practice in spite of concerns about frequent severe, dose limiting toxicities. Next, we introduce the development of targeted treatment as a concept for advancement within the broader field of small-molecule drug combination therapy in cancer and its accelerating progress that was boosted by recent scientific and technological progresses. Finally, we describe an alternative strategy of drug combinations using drug-conjugates for selective delivery of cytotoxic drugs to tumor cells that potentiates future improvement of drug combinations in cancer treatment. Overall, in this review we outline the development of chemotherapy from a pharmacological perspective, from its early stages to modern concepts of using targeted therapies for combinational treatment.

## 1. Introduction

Recorded attempts to cure malignant diseases date back to ancient times. However, modern chemotherapy is about 100 years old and in an almost poetic sense, its beginning could be entitled: “From chemical warfare to cancer therapy”. Indeed, many attribute the birth of modern chemotherapy to the introduction of sulfur mustard gas as a chemical weapon in World War I (WWI), which subsequently led to extensive military research in the pre-World War II (WWII) era and the discovery of its antiproliferative properties [1]. The lethal properties of mustard gas to cells were found to be directly proportional to the rate of cell proliferation. This physiological feature of mustard gas led to the implementation of mustard nitrogen as the first anticancer medication [2]. Mustard nitrogen is a DNA-alkylating agent that represents the first generation of anticancer drugs; toxic agents which are active against all proliferating cells, including normal ones. In this category of drugs also included are DNA intercalators and topoisomerase inhibitors, which were discovered and introduced into the clinic in the mid-1960s. The discovery that cancerous cells are a preferable target for toxic agents was encouraging, yet the overall excitement was restrained, since in a vast majority of cases the therapeutic effect of mustard gas derivatives on cancer patients was transient and patients typically succumbed to the disease shortly after cancer recurrence. Since the first use of mustard nitrogen as an anticancer medication, the evolution of chemotherapy was driven by the development of new laboratory research tools and accumulating knowledge about cancer diseases. The more selective approach of targeting special metabolic needs of cancerous cells relied on an understanding of the hypermetabolic activity of these cells. The first antimetabolite was introduced in 1948 by Sidney Farber and coworkers, who used the folate antagonist aminopterin for the treatment of childhood acute lymphoblastic leukemia (ALL) [3]. Shortly after the introduction of antifolates, a different class of antimetabolic drugs—purine and pyrimidine analogs such as 6-MP and 5-FU were discovered by a rational approach of target-oriented drug discovery [4,5]. Despite the discovery and clinical implementation of new anticancer drugs, the outcomes of single-drug treatments were still very poor, which brought about quick recognition that treatments with multiple drugs are necessary for the improvement of cure rates. Not without strong objections and skepticism from the oncology community, initial drug combination trials took place in the early 1950s, first in childhood acute lymphoblastic leukemia (ALL), and remarkable success of this approach led to its translation to other types of cancer down the road. Of note, the drugs that were used in the early drug combination clinical trials in childhood ALL during the 1950s and 1960s are still part of the foundation of contemporary pediatric ALL treatments with survival rates of about 90%.

Increasing understanding of complex cancer biology on a molecular level and the development of genetic analysis tools enabled the substantiation of the “magic bullet” concept—a 100 years old idea suggested by P. Ehrlich according to which cancer cells are selectively targeted by potent drugs while sparing normal tissues [6]. The “targeted therapy era” in cancer chemotherapy was manifested by the discovery of imatinib. Introduction of imatinib revolutionized the treatment of patients with BCR-ABL1 driven chronic myeloid leukemia (CML), increasing long-term remissions from about 20% before 1990 to more than 90% today [7] and during the last three decades, the focus has sharply moved from the development of cytotoxic drugs to molecularly targeted agents.

The technological leap of high throughput techniques and the beginning of the “omics” era in biomedical research enabled cancer researchers to acquire and analyze massive volumes of genomic, transcriptomic, proteomic and metabolomic data in short periods of time [8,9]. This accelerating accumulation knowledge enabled the identification of new molecular targets for selective cancer therapies and has hence supported the development of combinational targeted therapy in cancer. The ability to inhibit multiple oncogenic pathways simultaneously using these new types of drugs broadens the overall scope of combinational drug treatment and is a promising strategy to tackle the elusiveness of cancer, which relies on its ability to recruit and activate compensatory survival pathways or develop acquired resistance due to clonal evolution. The discovery of the CRISPR-Cas9 gene editing technique facilitated the ability to conduct high-throughput whole-genome screening experiments, which offers an unprecedented opportunity in oncology drug discovery; expedited identification of synthetically lethal targets per cancer type as a basis for rational drug combinations [10,11].

The purpose of the present review is to outline the historical development of chemotherapy from its beginning as a therapy based on single cytotoxic agents to the evolved combinational drug approach which includes the use of targeted therapies and is driven by advanced technology and accumulation of scientific knowledge. We describe the discovery processes of the main chemotherapeutic drug families and their contribution to the development of combinational drug treatments. During the course of this review we touch upon the key scientific breakthroughs that were directly contributing and supporting preclinical research that enabled the progress of combinational drug therapy in cancer. Finally, with discuss several experimental approaches such as chimeric drug entities and combinational targeted drug delivery (TDD) as potential platforms for future development and improvement of drug combinations in cancer.

## 2. The Origins of Modern Chemotherapy

### 2.1. Pre-Modern “Oncology” and Early Attempts to Implement Arsenic Compounds for Cancer Therapy 

**Figure 1:**
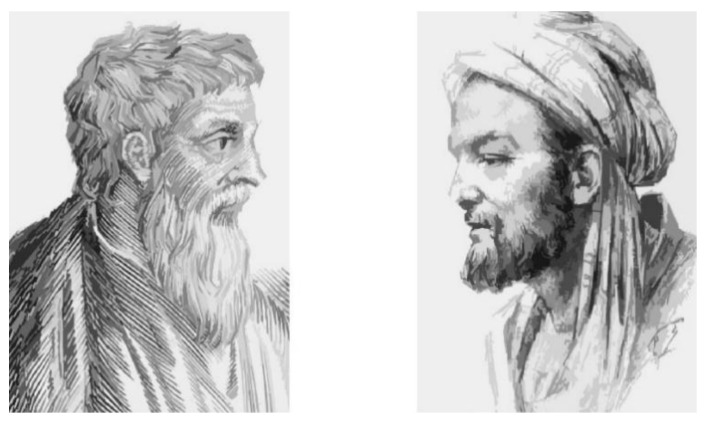
The practice of using chemicals to treat cancer traces back to ancient times. Dioscorides (**left**) mentioned the application of colchicine for cancer treatment in his comprehensive pharmacological encyclopedia, *De Materia Medica*, around 70 C.E. More than 800 years before Lissauer discovered the anticancer potential of Fowlers solution, the Persian physician Avicenna (**right**) implemented arsenic as an anticancer remedy.

Clinically effective chemotherapy was established as a key treatment option in modern oncology less than one hundred years ago. Nevertheless, the use of chemicals for cancer treatment traces back to ancient times. Many of today’s anticancer drugs and drug candidates originate from the same natural sources, as occurred hundreds of years ago, mostly from plants. For example, the use of colchicine for cancer treatment, which was first isolated and described as an antimitotic agent in the 19th century [12,13,14], actually dates back to 1st century A.D: “an extract of the autumn crocus, when soaked in wine and ingested could dissolve tumors (oidemata) and growths (phumata) not yet making pus” (Dioscorides (Figure 1, right); “*De materia medica*”). For further reading about ancient therapies see in *Ancient and Medieval Chemotherapy for Cancer*—John M. Riddle [15]. Another example of chemicals that long have been known for their anticancer properties are arsenic compounds: these also are the first nonherbal anticancer agents. The “double edged sword” nature of these compounds, as both poisons and therapeutics, has been known for thousands of years [16,17,18]. The implementation of arsenic compounds in medicine was broad and took place in different parts of the globe and civilizations [18,19]. The first recorded use of arsenic as an anticancer remedy belongs to the famous Persian physician Ibn Sina (Avicenna) (Figure 1, left) in 11th century C.E.

**Figure 2:**
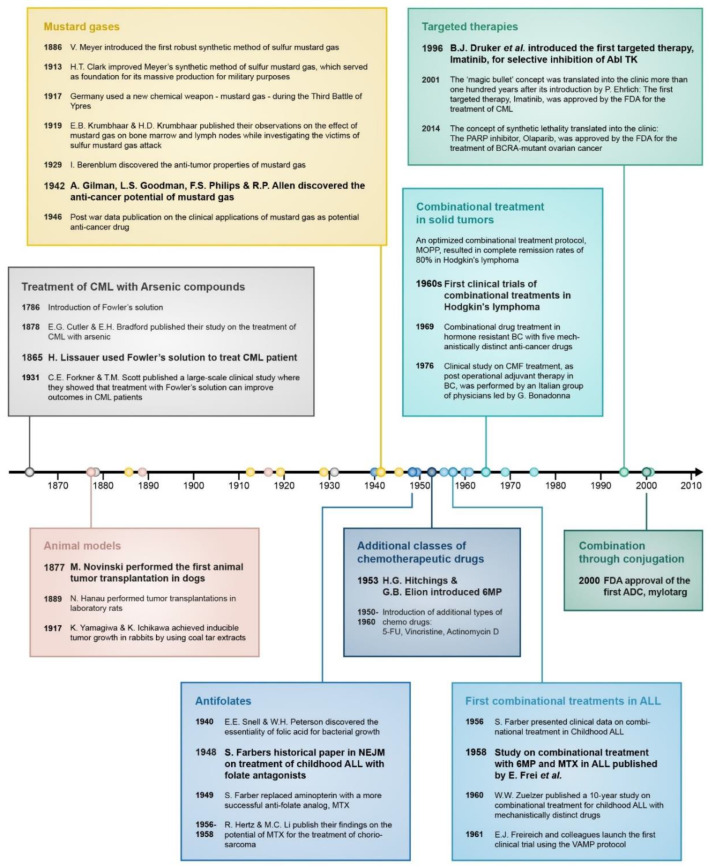
Timeline of important events that contributed to the development of drug combination treatment in cancer. In bold are the most significant breakthroughs in each category.

While being recognized for their therapeutic properties, the toxic nature of arsenic compounds narrowed their medical implementation, and they were used sparingly. In 1786, English physician Thomas Fowler introduced what was latter called “Fowler’s solution”—a remedy that contained 1% potassium arsenite, which was very popular in treatment of various ailments for more than a century (Figure 2) [20]. In 1865, German physician Heinrich Lissauer used Fowler’s solution for the treatment of chronic myelogenous leukemia (CML) [21]. However, the success of this treatment was limited, and Lissauer’s patients succumbed to their disease in a short period of time. Nevertheless, for the first time it was demonstrated that a transient relief from a malignant disease could be achieved by a toxic chemical. Though Lissauer did not discover the use of arsenic compounds for cancer treatment [1], he independently rediscovered its anticancer properties, establishing the critical concept that a chemical could function as an anticancer agent. In 1878, thirteen years after Lissauer’s finding, Cutler and Bradford published a more detailed study, where they showed that treating CML patients with potassium arsenite results in a decreased white blood cell count [22]. However, due to only having a temporary benefit, a lack of statistical confidence in these clinical reports and the emerging realization for the effectiveness of Roentgen’s X-ray treatment led to the abandonment of arsenic compounds for cancer treatment for decades. In 1931, Forkner and Scott renewed attention to Fowler’s solution, with a larger-scale clinical study in which they showed that administration of Fowler’s solution brought about an improvement in 9 out of 10 patients with CML [23]. Subsequently, potassium arsenite became a first-line standard of care for CML along with irradiation, until the introduction of the alkylating agent busulfan in 1953. The use of arsenic compounds as chemotherapeutics enjoyed a renaissance in the mid-1990s with the discovery of arsenic trioxide (As_2_O_3_) as a therapeutic for acute promyelocytic leukemia (APL) [24] and its subsequent successful use in the clinic [25]. Since then, arsenic trioxide is a standard of care for this type of malignancy in combination with retinoic acid [26,27,28].

### 2.2. Establishment of Animal Models for Studying Toxic Compounds 

Early clinical studies on the implementation of arsenic compounds to treat liquid malignancies foreshadowed the establishment of modern chemotherapy. The ability to induce and transplant cancers in animal models provided a reliable experimental platform, which was necessary for systematic studies of new chemotherapeutic candidates. The first successful transplantation of a tumor from one animal to another was demonstrated in 1877 by M. Novinski in dogs [29,30,31] followed in 1889 by N. Hanau in rats [30]. Introduction of the first inbred strains of mice at the beginning of 20th century [32,33,34,35] was the basis for reproducible experimental results. In 1917, two Japanese scientists Yamagawa and Ichikawa published their findings on the induction of tumors by coal tar in rabbits [29]. This pioneering work led to identification of the actual carcinogen component in the coal tar, benzo[a]pyrene. Subsequent isolation of benzo[a]pyrene [36,37] contributed to the production of related synthetic analogs, which enabled the feasible induction of carcinogenesis in experimental animal models. Another key development in the establishment of in vivo models for tumorigenesis came from the use of immunodeficient athymic Nude mice and severe combined immunodeficiency (SCID) mice as host animals able to serve as recipients for human tumor cell lines [38]. Decades later, the introduction of patient-derived xenograft (PDX) models, where human tumor tissue can be directly injected into immunodeficient mice, has expanded the range of possibilities of animal models for cancer drug research [39,40].

### 2.3. Mustard Gasses—The Beginning of Modern Chemotherapy 

Ironically, it was infamous chemical warfare that served as an accelerator for anticancer drug discovery. At the end of 19th century, chemical synthesis and production of mustard gas were intensively explored for military purposes. First reports describing the chemical synthesis and irritating properties of mustard gas track back to the mid-19th century [41,42]. In 1886, Viktor Meyer introduced a robust method for the synthesis of sulfur mustard gas [43,44], and in 1913, Hans Thacher Clarke, who was an expert in the chemistry of organic compounds of sulfur, optimized Meyers’ method [45]. Clarkes’ process served as foundation for an extensive military research by Germany, which subsequently implemented mustard gas as a chemical weapon in WWI [46].

**Figure 3:**
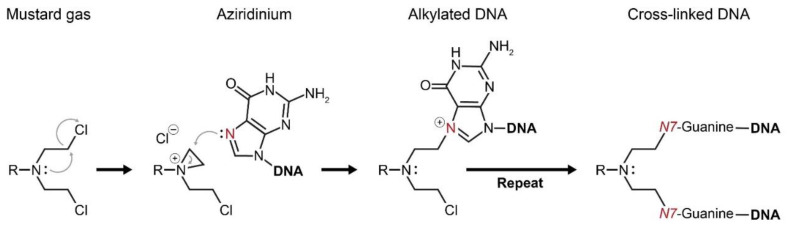
Mustard gases form a highly reactive intermediate—aziridinium. Aziridinium attracts functional groups of proteins and DNA to react with it, which eventually brings about cellular damage. Alkylation of the DNA base guanine by aziridinium results in a cross-linked DNA double strand. Consequently, cellular division cannot occur and the DNA damage results in apoptotic cell death.

Mustard gas causes severe cellular (Figure 3) damage, and its impact on bone marrow and the hematopoietic system was first described by Edward and Helen Krumbhaar; during WWI Dr. Edward Bell Krumbhaar was a medical officer who served with the American expeditionary forces in France along with his wife, Helen Dixon Krumbhaar. While investigating the victims of German sulfur gas poisoning at the Third Battle of Ypres, the couple Krumbhaar found that exposure to the agent resulted in depletion of bone marrow and reduction of lymph nodes [47,48]. In 1929, while studying carcinogenesis, Isaac Berenblum tried to promote tumor formation on mouse skin by adding sulfur mustard gas to carcinogenic tar, but he discovered a “diametrically opposite” phenomena; the mustard gas possessed anticarcinogenic properties [49,50]. Inspired by Berenblums’ observations, Frank E. Adair and Halsey J. Bagg performed additional and more comprehensive studies in animal models, demonstrating the anticancer potential of mustard sulfur [51].

WWII stimulated additional research on poisonous chemicals and the U.S. Office of Scientific Research and Development (OSRD) funded research at Yale University to support the development of chemical warfare [1,52]. For this mission, two young pharmacologists, Alfred Gilman and Louis S. Goodman, were recruited and in close collaboration with F.S. Philips and R.P. Allen, they investigated the biochemical properties of sulfur and nitrogen mustard agents. Early studies on animal models [53,54,55] led Gilman and Goodman to question whether it was possible to destroy a tumor with this group of cytotoxic agents before destroying the host [53]. To test this hypothesis, T. Dougherty from the department of anatomy joined the research team, and successful experimental results led to the first clinical trial, which was performed by G.E. Lindskog in 1942: A patient with an X-ray-resistant lymphosarcoma was subjected to intravenous administration of nitrogen mustard, which resulted in complete remission of the disease. Though the relief was transient [56], this single-patient clinical trial is a milestone in the history of chemotherapy, since it proved the concept that complete remission of advanced malignancy can be achieved by systemic administration of a cytotoxic drug [2]. Publication of these results, along with the publication of similar observations by other research groups [2,53,57,58] was only possible in 1946, after the ban associated with wartime data classification was removed, and it is considered by many as the birth of modern chemotherapy. One of the key publications during the post-WWII period on the clinical applications of mustard nitrogen was by Leon O. Jacobson and his group, where they introduced mustragen, which was the first nitrogen mustard with broad clinical application [59]. First-generation nitrogen mustards, namely mustragen and (tris(2-chloroethyl)amine) are no longer used in clinic. Instead, less toxic derivatives with higher pharmacological indexes, including prodrug-like analogs such as bendamustine and estramustine, were developed. During the ongoing development process of alkylating agents since the 1940s, several new classes of alkylating agents emerged [60], while the discovery of the antineoplastic activity of platinum-based compounds in 1965 [61] was probably the most important breakthrough. The clinical importance of the discovery of platinum-based drugs is ultimately demonstrated by the fact that about 50% of all cancer patients are treated with this class of drugs [62].

### 2.4. Antifolates and Nucleoside Analogs

Antimetabolites are chemicals that have structural similarities with endogenous metabolites. As opposed to metabolites, which are essential for cellular metabolism, antimetabolites interfere with normal metabolism by competing with metabolites for binding to enzymes, or through dysfunctional incorporation into final biosynthetic products. For instance, analogs of nucleic acids, which can be utilized as “false” nucleosides, have both capabilities. They are able to inhibit the enzymatic activity of DNA polymerases and also can lead to formation of malfunctioning biosynthetic products through incorporation into a replicating DNA strand [63,64].

**Figure 4:**
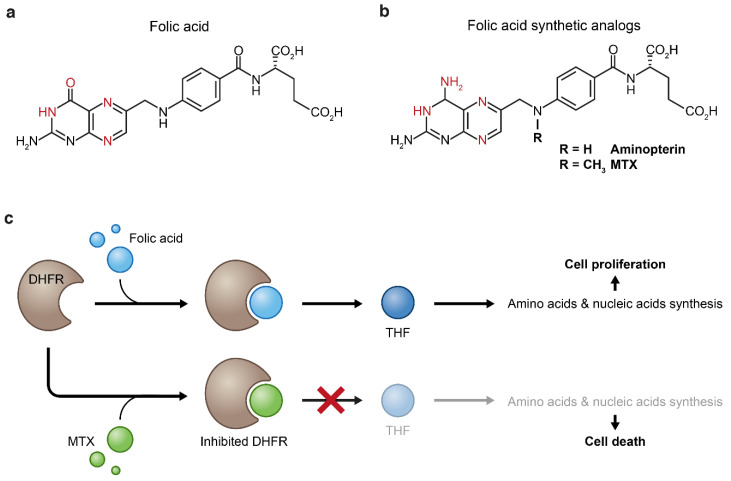
Folic acid (**a**) and its synthetic analogs (**b**). Both folic acid and its analogs bind to dihydrofolate reductase (DHFR) through formation of hydrogen bonds (the atoms that form these bonds are in red). Replacement of the enol group of folic acid by an amine group results in increased binding affinity of the synthetic analogs to the DHFR enzyme which inhibits the biosynthesis of tetrahydrofolate (THF) (**c**). Tetrahydrofolate starvation causes impaired cellular anabolism which eventually leads to cellular death.

Basic research that led to the discovery of pteroylglutamic acid as an essential metabolite in the early 1940s enabled successful clinical application of its structural analogs for targeting folic acid metabolism in childhood ALL; In 1940, E.E. Snell and W.H. Peterson published a pioneering study, where they reported that a factor (or factors) in a norite filtrate was able to stimulate the growth of *Lactobacillus casei* [65]. Accordingly, they named this factor “norite elute”, which was later named pteroylglutamic acid, and described it as a “showing some properties in common with naturally occurring purines”. In a follow-up paper in 1941, Peterson and his team made a few additional insights into the chemical-physical properties of this factor, including negation of its identification as a nucleotide, as was suggested by E.L.R. Stokstad [66]. They also showed that the same factor is essential for the growth of another species, *Streptococcus lactis* [67]. The biological importance of pteroylglutamic acid led to intensive chemical research, resulting in its first successful synthesis. This study was published as a short report by R.B. Angier et al. in 1945 [68], and in a series of subsequent follow-up studies, the structure of the molecule was resolved [69,70,71,72,73]. The common name of pteroylglutamic acid is “folic acid” and it was coined in 1941 from *folium* (leaf in Latin), since the substance is abundant in green leaves [74]; the substance contains a hetero-cyclic pteroyl moiety, and therefore shares physical and structural similarities with purines (Figure 4a,b). The essentiality of folic acid for rapidly proliferating organisms, along with observations by Lewisohn et al. that ‘‘folic acid concentrate’’ brings about regression of breast cancer (BC) in mice [75] and the newly established capability to synthesize its antagonists, made it possible to introduce antifolates into the clinic as first-in-class antimetabolic agents. The results were published in 1948 by Sidney Farber and his associates, in a study that is considered as one of the cornerstones of modern chemotherapy [3]. Farber’s simple assumption was that administration of false folate molecules will block normal folate supply to rapidly dividing cancer cells and stop their uncontrolled growth (Figure 4c). Indeed, administration of the folic acid antagonist, aminopterin (Figure 4b), to children with ALL resulted in clinical improvement—an observation that was supported by others in following studies a year later [76,77]. This seminal study was the first evidence that the proliferation of cancer cells can be halted by antimetabolites. Like the vast majority of anticancer drugs, the beneficial therapeutic effect of aminopterin was accompanied by high toxicity [78]. Therefore, aminopterin was replaced by a more effective analog, methotrexate (MTX) (Figure 4b), which was first synthesized in 1947 [79,80] as part of intensive attempts in the mid-end 1940s to synthesize more effective antifolates, and clinically used in a subsequent Farbers’ study reported in 1949 [81].

Following the successful implementation of antifolates in cancer therapy, primarily in blood malignancies, purine and pyrimidine analogs that represent other type of antimetabolic compounds, were discovered. The introduction into the clinic of the first purine and pyrimidine analogs, 6-mercaptopurine (6-MP) and 5-fluorouracil (5-FU), in the 1950s was the beginning of a continuously growing variety of this drug family and the ongoing optimization of its applications in cancer treatment. Along with the more recently discovered antimetabolites such as cytarabine, fludarabine and gemcitabine, the prototypical antimetabolic drugs, including MTX, 6-MP and 5-FU, are still standard components of chemotherapeutic treatment in wide range of cancers [82].

### 2.5. DNA Intercalators

**Figure 5:**
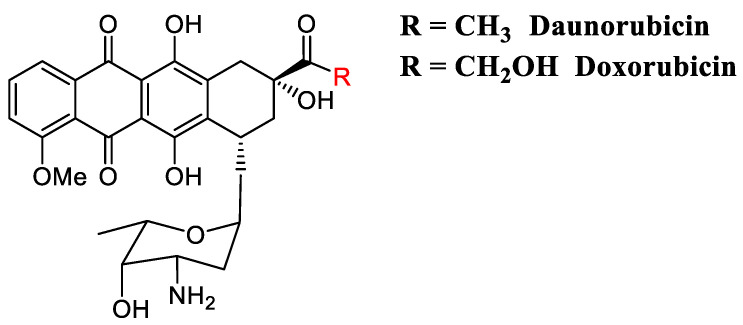
Daunorubicin and doxorubicin are amongst the most active and extensively used anticancer anthracycline-intercalators used in clinic.

DNA Intercalators are a unique class of biologically active molecules that are usually characterized by planar-polycyclic-aromatic structure with positive charge. Due to their physical features, intercalators easily insert between the planar bases of dsDNA through π-stacking, van der Waals bonds and hydrophobic interactions, resulting in DNA damage and interference with DNA replication [83]. The intercalator-DNA complex is stabilized by electrostatic interactions between positive charges on the intercalator and the negatively charged phosphate backbone of DNA [84,85,86]. Stable DNA-intercalator complexes may lead to inhibition of DNA unwinding enzymes Topo I and Topo II [87,88,89,90], and subsequent DNA damage-induced apoptotic cell death [91]. The fact that intercalators interfere with a basic biological process which is crucial for highly proliferating cancer cells but is silent in most normal tissue cells led to their consideration as highly potent chemotherapeutics. Since the discovery of the DNA intercalation process by L.S. Lerman and his coworkers in 1961 [92], many small-molecule intercalators, both naturally occurring and synthetic, have been evaluated as potential anticancer agents [93,94]. Currently there are several intercalators approved by the FDA for the treatment of different malignancies [83]: Actinomycin D is a bacterial-origin antibiotic that was discovered by S.A. Waksman and H.B. Woodruff in 1940 [95]. Due to its antiproliferative activity, actinomycin D was approved by the FDA in 1964 as a first-in-class anticancer intercalator drug [96]. Following the discovery of actinomycin D, several other antibiotics were isolated from bacteria and found to be potent anticancer agents. Daunorubicin and doxorubicin (Figure 5) are the most representative examples of bacterial-origin anthracycline-intercalators that have been approved as anticancer agents. Since their discovery in the early 1960s and subsequent introduction into the clinic, daunorubicin and doxorubicin are amongst the most active and extensively used anticancer agents [97]. The discovery of intercalating agents as a new class of anticancer drugs significantly contributed to the diversification of combinatorial chemotherapy and enabled improvement of clinical outcomes for hard-to-cure malignancies, such as advanced BC [98,99]. However, the toxicity that is associated with anthracycline chemotherapy, in particular cardiotoxicity [100,101], ultimately limits their clinical application and their use is becoming restricted almost exclusively to high-risk patients [102,103,104].

## 3. Combination of Drugs as Strategy to Achieve Better Clinical Outcomes

### 3.1. Drug Combinations in Childhood ALL 

First-generation anticancer drugs that were discovered in the mid-20th century, in major part through research into leukemias [105], changed the landscape of clinical oncology. However, the benefit of these drugs was typically transient, and cases of complete cure were rare. Even in cases of nonrefractory malignancy, where strong initial responses to therapy were observed, aggressive recurrent disease occurred that commonly was no longer responsive to treatment. The evolutionary model of cancer, where genetically distinct tumor cell subpopulations possess genetic alterations, was thought to be responsible for cancer drug resistance. Under the selective pressure of chemotherapy, subpopulations of cancerous cells expand to become the dominant surviving population [106]. This still is thought to be one of the reasons why single agent-based therapy is usually not sufficient to achieve a complete cure. Improved clinical outcomes that were achieved by attacking genetically distinct *Mycobacterium tuberculosis* cells with drug combinations [107], as well as pioneering studies in animal cancer models that reflect tumor cell genetic heterogeneity [108,109,110,111], provided experimental support for the assumption that combinational chemotherapy can significantly improve clinical outcomes. Co-administration of cytotoxic drugs is thought to target different pro-oncogenic pathways that can be active simultaneously in a tumor cell. By that means, drug combination therapy deprives tumor cells of compensative survival mechanisms.

The establishment of combinational chemotherapy as a standard of care in modern clinical oncology was possible due to a persistence of intrepid physicians and dedicated and inspired medicinal chemists. The groundbreaking studies of S. Farber on the treatment of childhood ALL were executed through close collaboration with the remarkable medicinal chemist Yellapragada Subbarow. Subbarow and his team at Lederle Laboratories provided the necessary supply of folate antagonists for Farber’s clinical investigations and were major contributors to the development of synthetic methods of antifolates. The discovery of MTX was part of these pioneering chemical research efforts on antifolates, and it has a special place in the history of chemotherapy as being the first single drug that enabled to cure a solid tumor: Two scientists from the national institute of health (NIH), R. Hertz and Min C. Li, went on to show that MTX can be used to completely cure choriosarcoma [112]. In a follow-up paper, Hertz and Li also showed that combinatorial treatment with MTX and 6-mercaptopurine (6-MP) could dramatically improve outcomes for fatal gestational trophoblastic disease (GTD) [113]. MTX was also a pivotal component for the evolving clinical investigation of combination chemotherapeutic treatment. H.G. Hitchings and G.B. Elion, two distinguished medicinal chemists who worked together for many years, significantly contributed to the development of medicinal chemistry as a branch of synthetic organic chemistry, establishing the concept of “rational drug design” [4,5]. One of many remarkable outgrowths of their work was the synthesis of a purine antagonist 6-MP [114,115,116], which was another essential pillar in the early stages of combination chemotherapy.

Oncologists such as E. Frei, E. J. Freireich, V. DeVita, J. H. Burchenal and D. Pinkel ceased to favor single agent-based treatments. Driven by strong rationale and empirical evidence that drug combinations can substantially improve cancer cure rates, they launched pioneering clinical investigations in the early 1950s to extend upon these findings. In a comprehensive report on the status of chemotherapy in 1956, S. Farber discussed clinical data concerning the use of combinational antimetabolite therapy, either MTX or 6-MP, with glucocorticoids in childhood ALL, pointing to its advantage over single agent-based treatments [117]. In 1958, E. Frei et al. published a study on fine-tuning the scheduling and doses of combinational treatment with MTX and 6-MP to achieve optimized outcomes in ALL [118]. In 1950, Wolf W. Zuelzer and coworkers launched a 10-year clinical study in which three mechanistically distinct chemotherapeutics were utilized [119]. This study was done on a group of children with ALL and was the first clinical report of using three chemotherapeutic agents with distinct mechanisms of action [119]. Zuelzer applied a therapeutic regimen that he later named “composite cyclic therapy” (CTC) [120], which comprised a combination of steroids (cortisone, prednisone or dexamethasone), antimetabolites (aminopterin, amethopterin or 6-mercaptopurine) and alkylating agents (melamine or nitrogen mustard). The early success of these chemotherapeutic treatments, especially in childhood ALL, stimulated drug discovery research at the federal level in the United States, as manifested by the establishment of the Cancer Chemotherapy National Service Center (CCNSC) in 1955 [121]. At the same time, the growing potential of the anticancer drug market encouraged pharmaceutical companies to invest more in research. One of the first fruits of commercial drug discovery was the introduction of a new class of antineoplastic drug, vincristine, by the Eli Lilly Company. Vincristine was approved by the FDA in 1963 [122] and, unlike most other chemotherapeutics, it does not target the DNA, but rather arrests mitosis by targeting the cytoskeleton. Addition of vincristine to a narrow repertoire of anticancer drugs of that time increased the versatility of possible drug combinations and contributed to an effective combination treatment for childhood ALL [123]. This culminated in the development of a successful quadruple combination, comprised of vincristine, amethopterin, 6-MP and prednisone—known as “VAMP” [124].

With the polydrug regimen that was developed in early 1960s still at the backbone of treatment, the overall 5-years survival rates of childhood ALL today are exceeding 90%—while in mid-20th century this disease was 100% incurable. Impressive clinical outcomes of drug combinations in childhood ALL were a cornerstone of drug combinations in cancer treatment in general, and this has been extended to other malignancies. Today there are several ongoing clinical trials of frontline combinational chemotherapies with targeted drugs, such as the proteasome inhibitor bortezomib, the JAK inhibitor ruxolitinib and the PI3K/mTOR inhibitor NVP-BEZ235, which are mainly aimed to achieve better responses in adults for relapsed/refractory ALL [125].

### 3.2. Expanding Combination Drug Therapy to Additional Cancer Types

Using drug combinations with adjusted doses and careful optimization of administration scheduling represented a successful translation from preclinical animal models into the clinic, resulting in increased remission rates in childhood ALL. For further optimization of drug combination strategies and for expanding its application to other cancers, these principles were applied in the mid-1960s to treat Hodgkin’s lymphoma. The first pilot study was inspired by the success of the VAMP protocol in childhood ALL [124]. The study included vincristine, which was used in combination with cyclophosphamide, prednisone and MTX [126,127,128]. As folic acid antagonists possessed very limited efficacy in the treatment of lymphomas [129], MTX was later replaced by procarbazine, which by itself was showing potent activity against Hodgkin’s lymphoma [128]. Further optimization of this combination was achieved by replacement of cyclophosamide with another nitrogen mustard—mustragen, resulting in the “MOPP” protocol (Mustargen/mechlorethamine, Oncovin/vincristine, Procarbazine and Prednisone). The MOPP protocol enabled complete remission rates in up to 80% of patients with advanced Hodgkin’s lymphoma, and was used as the standard of care for this disease for more than 20 years, until it was replaced by other, even more successful and less toxic protocols [130].

Examples of single-agent successes such as the cure of choriosarcomas with MTX treatment alone are rare exceptions. The flexible and modular nature of drug combination therapy enabled its continuous optimizations and further adaptation to other solid tumors. In 1969 Cooper et al. obtained encouraging results, showing a 90% responsive rate in hormone-resistant breast cancer with a five-drug treatment regimen (cyclophosphamide, methotrexate, fluorouracil (5-fluorouracil [5-FU]), vincristine sulfate and prednisone) [131,132]. This protocol was then subjected to modifications by the original authors [131] as well as other groups [133,134] to mitigate adverse side effects of this multidrug treatment and improve clinical outcomes. At that time, a combination drug treatment for BC, called CMF (cyclophosphamide, methotrexate and fluorouracil), was investigated to address the need for postoperative adjuvant treatment, since the vast majority of patients were expected to relapse after surgery. These studies were subsidized by the National Cancer Institute (NCI), but due to lack of enthusiasm among BC clinicians in the US at the time [135], much of this work was performed by G. Bonadonna and his group in Italy; it resulted in improved cure rates and ultimately led to widespread acceptance for this form of treatment [136]. This international collaboration resulted in drug-combination protocols that remained a standard of care in BC for many years and served as the foundation for present-day BC adjuvant therapy.

### 3.3. Targeted Therapies—The Expanding Universe of Drug Combinations

New discoveries and accumulating biological knowledge enabled significant advances in anticancer drug development. The “magic bullet” idea was introduced by the prominent physician Paul Ehrlich more than 100 years ago to portray the concept of an agent that could selectively kill malignant cells without harming normal tissues [6]. Deeper understanding of biological mechanisms at a molecular level enabled to develop drugs for targeting specific cellular pathways, transforming the “magic bullet” idea into reality. The first successful targeted therapy, imatinib, was discovered in 1996 [137], which marked the beginning of the targeted cancer treatment era. Early clinical applications of imatinib took place in the late 1990s, followed by its FDA approval in 2001. Imatinib is a small molecule that specifically inhibits the product of the 9–22 reciprocal chromosomal translocation named the “Philadelphia chromosome” [138] that results in a formation of the BCR-ABL tyrosine kinase fusion gene. ATP-dependent hyperphosphorylation kinase activity of all three oncogenic isoforms of BCR-ABL, mostly but not exclusively results in CML [139,140]. Imatinib binds to the ATP binding pocket of BCR-ABL, thus abolishing its kinase activity. Since its discovery, imatinib is still one of the most striking examples of successful targeting small-molecule therapeutics. Since the remarkable clinical results in CML patients who were treated with imatinib and other tyrosine kinase inhibitors (TKIs) [141], the main focus of anticancer drug development is concentrated on rational design of molecules that can specifically interfere with known oncogenic factors. Development of new small molecule targeting therapies is a fertile field, and dozens of new drugs for various oncogenic targets have been developed in the last two decades. Other than protein kinase inhibitors, which are still a very popular target [142] with 48 FDA drug approvals as for 2019 [143], small-molecule targeted therapies for other protein families such as epigenetic regulators [144,145] and transcriptional factors [144] are being developed as well. Even transcriptional co-regulators, which were traditionally considered to be an undruggable class of proteins [146], are now candidates for targeted cancer therapy intervention [147,148]. In contrast to that seen for imatinib in patients with CML, only partial therapeutic benefits are seen for most targeted drugs. This suggests that the ability of cancer cells to utilize more than a single oncogenic pathway necessitates combinational treatment in order to achieve improved clinical outcomes, as was previously recognized for traditional chemotherapeutic agents. The addition of new targeted therapies to the existing arsenal of anticancer drugs substantially increased the options for finding effective combination treatments, either by using combinations between two targeted therapies or by combining targeted therapies with traditional anticancer chemotherapeutics [149,150].

### 3.4. Synthetic Lethality and Beyond

**Figure 6:**
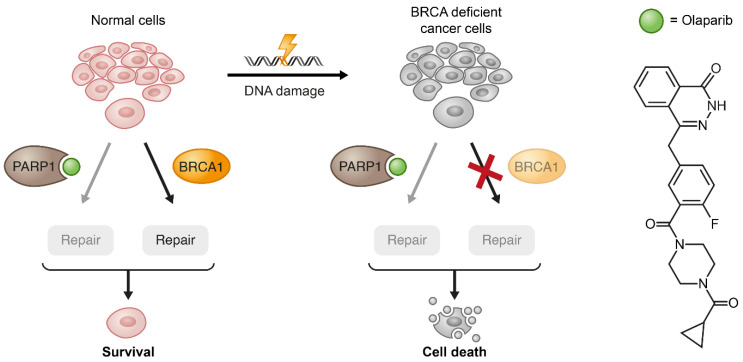
Exemplification of the synthetic lethality concept by PARP1 inhibition in BRCA1-deficient cancer cells. When the pharmacological inhibition of PARP1 combines with lack of wild type (WT) compensation mechanism (BRCA1) in cancerous cells, these cells become substantially more vulnerable to the treatment.

Synthetic lethality, as a concept when two or more genetic lesions cause lethality, originated from studies made in *Drosophila* almost a hundred years ago [151]. With the increasing ability of modern drug development to target specific biological molecular pathways, synthetic lethality studies have become a powerful tool for identifying strategies for killing genetically defective malignant cells while sparing normal tissues [11]. For instance, treating BRCA1/2-mutated tumors with PARP inhibitors is a classic example of taking advantage of a cancer-specific mutation to selectively kill cancer cells by depriving them of a biological mechanism (DNA damage repair) that compensates for this genetic deficiency (Figure 6) [152,153,154]. So far, the only clinical implementation of the synthetic lethality strategy is through the use of PARP inhibitors for patients with BRCA mutations. However, a set of recently developed techniques such as genome-wide screens and computational approaches are being successfully utilized for the discovery of new synthetically lethal interactions. For example, RNAi high-throughput screens revealed potential synthetic lethality targets in cancers with an aberrant expression of “undruggable” genes like KRAS [155,156] and cMYC [157]. Even in cases where direct inhibition of experimentally revealed hits is not possible, successful inhibition of pathways that are related to undruggable genes can be achieved by using targeted small-molecule inhibitors. For instance, using bortezomib and fasudil to inhibit GATA2-controlled NF-κB and RHO/ROCK signaling pathways, respectively, in the context of mutant KRAS in NSCLC mouse model resulted in impressive tumor regression [158].

**Figure 7:**
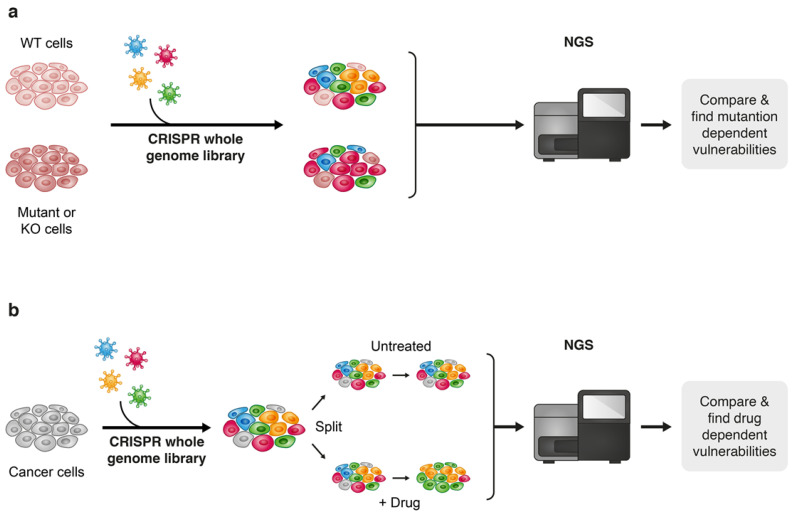
Two approaches towards discovering drug vulnerabilities using CRISPR-Cas9 genome-wide screens: vulnerabilities that are derived from a specific genetic contexts (**a**) and drug-related vulnerabilities (**b**).

Furthermore, next-generation sequencing (NGS) that enables feasible and fast analysis of the human genome, as well as other “omics” technologies such as proteomics, metabolomics and transcriptomics are playing a game changing role in the speed of understanding cancer at a high-resolution level. The “omics” era provides an unprecedented capability to accumulate massive volumes of data by high-throughput screenings of biological samples, and advanced computational tools enable to integrate this voluminous information, which creates fertile ground for the discovery of new cancer targets and therapies. Though the multilayer complexity of cancer and its ability to adapt to new conditions under therapeutic pressure still makes “the war on cancer” far from being complete, the ability to practically acquire and efficiently analyze massive amounts of biological information make the terms of this war less unequal. The “CRISPR revolution” presented a new technological platform that leverages the capabilities of NGS to enhance the performance of genome-wide high-throughput screens and since the pioneering publications of genome-scale pooled CRISPR-Cas screenings [159,160], this approach is now widely used to seek out the identification of new drug combinations for cancer treatment [10]. Though gene editing techniques such as TALEN and Zinc finger nucleases (ZFNs) [161,162,163] have been discovered before CRISPR, the straightforward approach of CRISPR-based gene editing established it as a key technique for broad genetic screens for the discovery of new drug combinations in cancer treatment [162,164]. There are two main approaches for drug discovery that implement the high-throughput CRISPR-Cas9 technology; one approach is to uncover genetic vulnerabilities within specific genetic contexts, the second is by performing the screen under pharmacological pressure of a known targeted therapy to reveal drug-related vulnerabilities and suggest potential effective combinations with the drug being evaluated (Figure 7). For example, Wei et al. found that sorafenib-resistant hepatocellular carcinoma (HCC) cells become highly sensitive to treatment when phosphoglycerate dehydrogenase (PHGDH) is knocked out. Subsequently, this finding led to the identification of an effective combinational treatment of HCC with sorafenib and a PHGDH inhibitor NCT-503 [165]. In another study, Szlachta et al. performed a CRISPR screen using a sgRNA library that covers nuclear genes, such as transcriptional and epigenetic regulators. The screen was performed under the pharmacological pressure trametinib in a patient-derived xenograft (PDX) model of pancreatic ductal adenocarcinoma (PDAC) to identify conditional lethality with MEK inhibition. The authors identified a cohort of cell-cycle and kinetochore genes whose knock out (KO) sensitized the tumors to treatment with trametinib. Validation of two of these genes, CENPE and RRMI, revealed that their pharmacological inhibition strongly synergizes with trametinib treatment across three different PDAC cell lines, as well as in an in vivo PDX PDAC model [166]. In a recent publication by Xu et al., the authors found through a genome-scale CRISPR-Cas9 screen that loss of function of a group of kinases improves the standard chemotherapeutic treatment of malignant pleural mesothelioma (MPM) with cisplatin and pemetrexed. Pharmacological inhibition of WEE1, the kinase whose KO possessed the most significant inhibitory effect on MPM cells, significantly increased the vulnerability of these cells to cisplatin/pemetrexed chemotherapy [167]. 

Overall, during the last decade there was a leap in the capabilities of new target discovery for combinational drug treatment in cancer, which was an ultimate result of technological progress and the discovery of the CRISPR-Cas gene-editing tool. These scientific advances are responsible for boosting drug discovery research in both academia and industry, and the fruits of these efforts in form of new therapies can be expected in the near future [11].

Beside the increased killing effect of cancer cells, additional beneficiary aspects of drug combinations can be realized, such as balancing of the immunosuppressive effects that are usually associated with chemotherapeutic treatment [168], and induction of immunogenic cell death to boost the immune attack on tumor cells [169]. Combinational treatments using small-molecule drugs with antibody therapies is an emerging treatment area in modern oncology, however, due to the limitation imposed by the scope of this review, we will only discuss in the next section the combination of small-molecules and antibodies as conjugated constructs. For further reading on combinational treatments with small-molecules and antibodies, readers are referred to outstanding reviews recently published on this theme [170,171,172,173].

### 3.5. Combining Through Conjugation—Implementing Antibodies, Peptides and Chimeric Molecules

**Figure 8:**
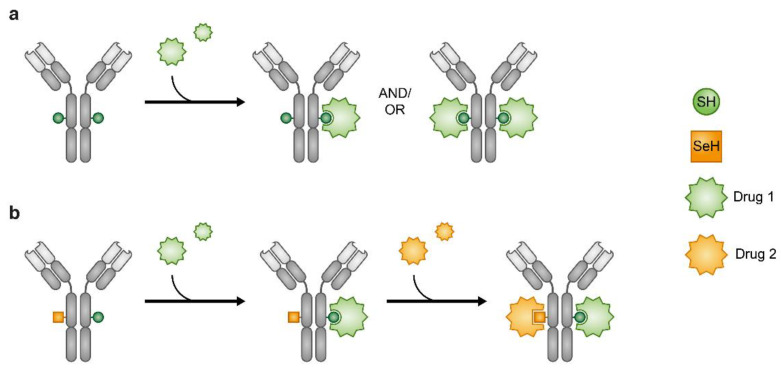
Antibody–drug conjugates (ADCs). Heterogeneous ADCs (**a**). Orthogonal reactivity of the SeH and SH enables production of ADCs where one Ab carries two different drugs (**b**).

Administration of drugs by their conjugation to carriers is an alternative approach to the use of traditional combination of drugs. Using antibodies (Ab) as carriers, to form antibody–drug conjugates (ADC) is a conventional method of drug conjugation. ADCs enable targeted delivery of toxic drugs to cancer cells, and their use is also an opportunity for delivering two distinctive therapeutic entities, the Ab and the small molecule conjugated to it, in a single construct. Since the FDA approval of the first ADC, gemtuzumab ozogamicin (mylotarg) in 2000, eight ADCs have been approved by the FDA as oncotherapies, while four of them have been approved during 2019–2020. Moreover, nearly 80 ADCs are currently in clinical development [174], which highlights the emergence of ADCs as a new class of therapeutic agents in current clinical oncology. However, similar to small-molecule anticancer drugs, acquired drug resistance has been observed in ADCs as well [175,176,177,178,179]. Therefore, similar to drug combination approaches with traditional small-molecule drugs, conjugation of warheads (drugs) with different mechanisms of action to a common carrier (Ab) has been proposed as a potential strategy to tackle the resistance problems faced with ADCs. The need for ADCs loaded with different drugs has necessitated the development of suitable chemical tools that were achieved by creation of orthogonally reacting linkage handles [180,181,182,183,184] and site-specific introduction of seleno-cysteine (Sec) instead of naturally occurring cysteine moiety into an Ab’s c-terminus [185,186]. The unique chemical reactivity of selenium produces two orthogonally reactive sites—Cys and Sec—that potentiate conjugation of different drugs to the same Ab (Figure 8) [186,187]. So far, ADC that is loaded with different drugs has not yet been clinically assessed. Nonetheless, the potential of ADCs in cancer treatment that is exemplified by the recent accelerated progress of this modality makes it an attractive subject for further clinical research with focus on the development of ADCs that carry mechanistically different drugs.

Peptides represent another type of carrier for drug combination through conjugation. Although Abs have some important advantages over peptides, such as superior pharmacokinetic properties [188] and the ability to penetrate the cell via endocytosis, their limitations such as narrow chemical diversity, bulkiness and immunogenicity, have made peptide–drug conjugates (PDCs) an attractive alternative [189]. Moreover, the superiority of peptides over Abs in key pharmacological parameters has motivated the development of approaches to improve their physiological stability and pharmacokinetic properties in order to close the gap between PDCs and ADCs [190]. Peptide cyclization, which limits the access of proteolytic enzymes, as well as incorporation of non-naturally occurring amino acids to prevent enzymatic recognition, are commonly applied for achieving longer stability of peptides in physiological environments [190]. Improved pharmacokinetic properties of PDCs are achieved by using biodegradable polymers [191], while enzyme-cleavable linkers are often used for tumor site-specific release of their cytotoxic cargo [192,193,194]. Many tumors overexpress one or more cell-surface markers like integrins [195], hormone receptors such as EGFRs [196,197], GPCRs [198] and immunoregulatory proteins [199,200]. Expression of surface markers on cancer cells provides an opportunity to selectively target them with peptides or peptidomimetics [201,202]. These cancer selective ligands (sometimes called tumor homing peptides) are usually, but not exclusively, implemented in a naked form as antagonists to their receptor molecules, and can also be used as carriers for targeted drug delivery (TDD) [203,204]. Currently there are no clinically approved PDCs, however, several PDCs have reached late stage clinical trials [205,206]. Approaches that implement peptides as platforms for drug combination, rather than just drug carriers, have been pursued as well: An analog of the integrin binding peptide, cilengetide [207,208], was incorporated into a PDC loaded with two mechanistically different chemotherapeutic drugs, chlorambucil (CLB) and camptothecin (CPT) [209]. Compared to the single drug conjugates (either CPT or CLB) this double-warhead PDC showed improved cancer cell killing effect in several solid cancer cell models. In another example, CLB and CPT were conjugated to a 13-meric myelin basic protein (MBP)-derived peptide to form a multi-warhead PDC for TDD in a leukemia cell culture model [210].

**Figure 9:**
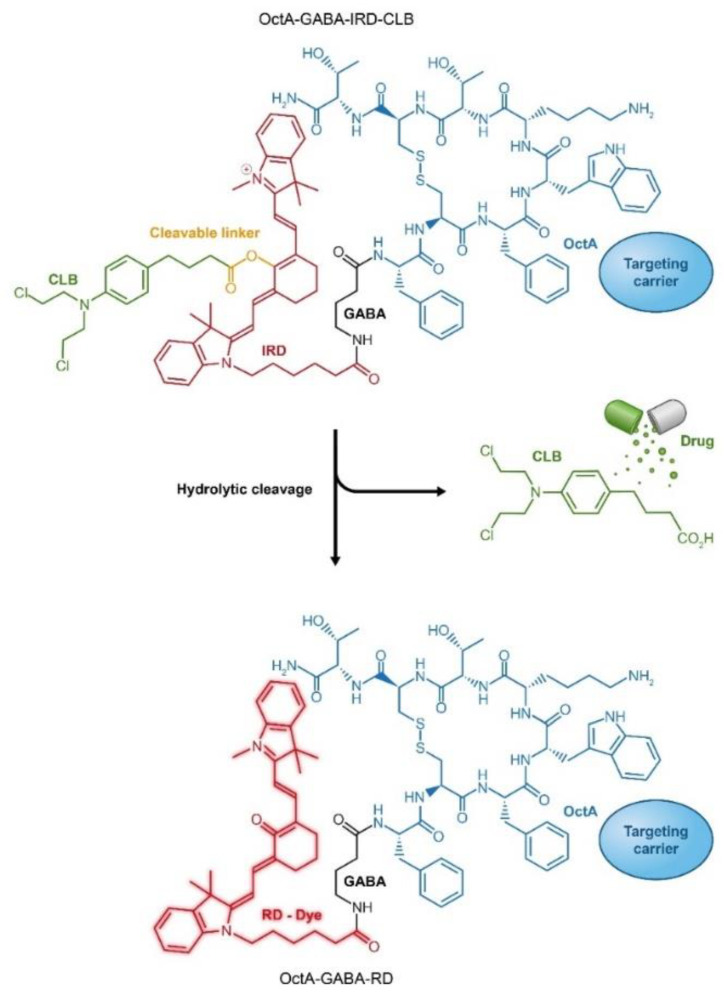
Targeted peptide carrier octreotide amide (OctA) loaded with an anticancer drug chlorambucil (CLB) through conjugation to a fluorescent dye IRD, which enables ratiometric measurements of drug release. RD, red dye; IRD, near infra-red (IR) dye.

Attachment of fluorescent reporters and cytotoxic drugs to the same carrier can be observed as a variation to combination through conjugation that exploits a single carrier for simultaneous loading with drug and fluorescent agent. By that means, in addition to the delivery of a cytotoxic drug to the cancer cell, tumor site(s) can be monitored. Several examples of such an approach have been demonstrated by using Abs as delivery platforms [211,212]. Alternative design of drug-fluorescent conjugates has been recently introduced using peptides as cancer-specific ligands. In this type of PDCs, the drug is attached to the carrier through a “switchable” fluorescent dye that changes its emission wavelength upon drug cleavage. Such design of drug-fluorophore PDC enables ratiometric monitoring of cytotoxic cargo release in the body during treatment (Figure 9) [213,214,215].

**Figure 10:**
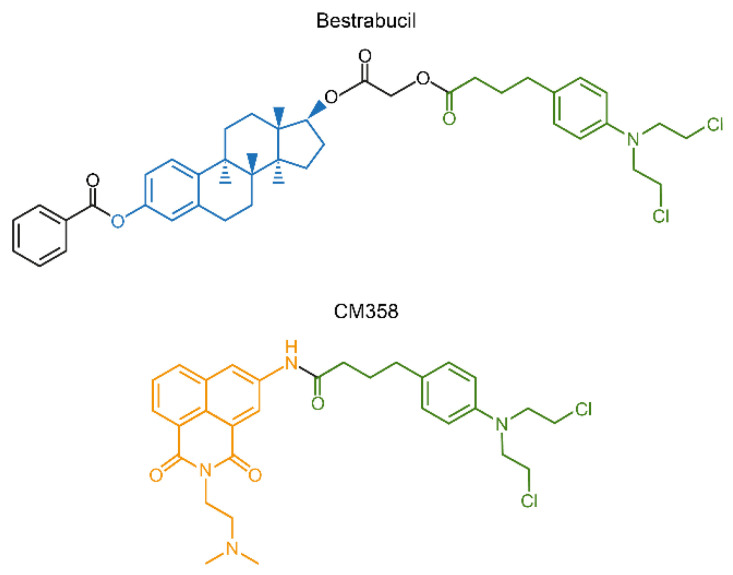
Chimeric compounds bestrabucil and CM358 are products of fusion of two different drugs into a single entity.

Another interesting approach for conjugation drug therapy involves direct fusion of two drugs into a single molecule. Bestrabucil (Figure 10) represents the first-in-class of such fused molecule achieved by the coupling of an estradiol derivative with CLB. Originally, bestrabucil was designed as a targeted agent with affinity for estrogen receptor-positive (ER+) tumors, but it was found to be active in several ER-negative cancers as well [216]. A similar approach involving direct attachment of two drugs to produce a single chimeric molecule was used to synthesize a small library of dimeric conjugates, each comprising two different chemotherapeutics. One of these chimeras, called CM358, is a fusion between CLB and the topoisomerase II (topo II) inhibitor amonafide (Figure 10). CM358 showed increased cytotoxicity in several cancer models as compared to either of its parent components, probably due to superior inhibition of the amonafide target topo II [217]. In another example, direct conjugation of irinotecan and CLB resulted in a dimeric chimera, which due to the hydrophilic-lipophilic properties of its components assembles into nanoparticles with improved PK/PD and increased antitumor activity [218].

## 4. Summary

Combinational drug treatment, developed as a branch of chemotherapy to address the issue of drug resistance, is ultimately becoming a standard practice in the clinic. Discovery of new classes of drugs contributed to the rapid expansion of drug combination possibilities, which has accelerated even more with the introduction of targeted small-molecule drugs. Targeted therapies have not only played a game-changing role in the treatment of various malignancies—in part it may meet the challenge proposed by the 120-year-old “magic bullet” concept—but it also substantially increased the range of options for combinational treatment. Synthetic lethality is another example of an old concept that has been successfully utilized in modern drug discovery for identifying effective anticancer drug combinations. Modern computational methods and the development of the revolutionary gene-editing tool CRISPR-Cas9 promise to be powerful engines for finding more effective drug combinations. New medicinal chemistry approaches designed to physically link multiple drugs together represents a distinct way to potentially achieve the benefits of drug combination for killing tumor cells with reduced toxicity to other tissues in the body. Since cancer can ultimately be thought of as a diverse collection of genetically distinct diseases, it is likely that no single drug or drug combination ever can be used to treat all cases. However, by adapting a range of anticancer drugs that target known oncogenic dependencies to a specific type of cancer, it ultimately might become possible to effectively treat and “approximate a cure” for more cancers with tailored drug combinations designed to cut off all tumor cell reservoirs of escape pathways.
